# Regenerative potential of CD200^-^ subpopulations of hair follicle bulge

**DOI:** 10.3389/fcell.2026.1808471

**Published:** 2026-04-23

**Authors:** Ting Sun, Sayuri Hamano, Jing Chen, Lichen Ling, Jinran Lin, Lei Yan, Tatsuto Kageyama, Fuyue Wu, Zheng Lin Tan, Wenyu Wu, Junji Fukuda

**Affiliations:** 1 Faculty of Engineering, Yokohama National University, Yokohama, Kanagawa, Japan; 2 ReMed Regenerative Medicine Clinical Application Institute, Shanghai, China; 3 Department of Dermatology, Huashan Hospital of Fudan University, Shanghai, China; 4 Kanagawa Institute of Industrial Science and Technology, Kawasaki, Kanagawa, Japan

**Keywords:** bulge, CD200, hair follicle regeneration, hair follicle stem cell, transcriptomic profiling

## Abstract

The hair follicle bulge is regarded as a reservoir of epithelial stem cells for hair regeneration. Hair follicle bulge cells are generally characterized by CD200 expression. However, the relationship between CD200^+^ bulge subpopulations and human hair regenerative capacity remains unknown. In this study, we systematically investigated the CD200- and KRT15-expressing human hair follicle bulge cells by integrating phenotypic characterization, transcriptomic profiling, and *in vivo* hair regeneration assays. CD200^-^ cells from freshly isolated human hair follicle bulge exhibited higher hair-regenerative capability. Using different culture systems, we have observed the correlation between reduced CD200 expression and increased hair regenerative capability of cultured hair follicle bulge cells. Our study refines the functional interpretation of CD200-defined bulge heterogeneity and provides insights for optimizing human bulge cell–based approaches to hair follicle regeneration.

## 1 Introduction

Stem cells are often characterized by their ability to differentiate into various cell lineage. Generally, they reside in their specific niche, where stemness is maintained (Blanpain et al., 2004). Interestingly, epithelial stem cells are often compartmentalized and comprise a heterogeneous population of cells that exhibit different transcriptional and anatomical profiles (Donati and Watt, 2015; Xin et al., 2016). While these stem cells contribute to tissue homeostasis and regeneration (Abbas and Mahalingam, 2009), they also possess plasticity to alter their own function when perturbed by appropriate signals.

Hair follicles, known for their self-renewal properties, cycle through three stages; anagen, catagen and telogen, and reconstitute throughout the lifetime as a reservoir of epithelial stem cells for both hair follicles and skin (Mistriotis and Andreadis, 2013). Within the hair follicle, there is a visible swelling structure at the outer root sheath known as “bulge”. The bulge located between the opening of the sebaceous gland and the attachment point of the arrector pili muscle (Ohyama, 2007). This region is also the permanent portion of the hair follicle, which exists regardless of the hair cycle.

The hair follicle bulge houses slow cycling hair follicle stem cells, which are usually considered quiescent due to their slow cycling properties (Cotsarelis et al., 1990). Discovery of slow cycling cells retention in the bulge has encouraged the proposal of “bulge-activation hypothesis”, which states that hair follicle stem cells in the bulge are activated in the late telogen stage, inducing regeneration of hair follicles in the anagen stage.

Analysis of the hair follicle bulge by immunohistochemistry suggested that this region is characterized by its expression of cytokeratin (KRT) 15, cluster of differentiation (CD) 200, KRT14, integrin β1 (CD29) and integrin α6 (ITGA6) (Inoue et al., 2009). Another study has also suggested that leucine-rich repeat-containing G-protein-coupled receptor 5 (LGR5) (Polkoff et al., 2022) and SRY-box transcription factor (SOX9) (Vidal et al., 2005) were also identified as markers of hair follicle stem cells.

Among these markers, CD200^+^ CD34^−^ hair follicle bulge cells are considered the dominant markers, and these cells possess a higher colony forming capability (Inoue et al., 2009). Although the reason is uncertain, CD200 has gradually become the marker used to identify hair follicle bulge cells (Jiang et al., 2010; Molina and Finol, 2020), particularly epithelial stem cells. However, it is also important to note that the bulge was originally defined as a region in the hair follicle outer root sheath. Therefore, CD200^+^ population might under-represent the various population of cells in bulge, as it indicate only a single population of it.

While various attempts have been made to regenerate hair follicles using CD200^+^ bulge cells, only murine hair follicle regeneration has been successful (Ohyama et al., 2008). However, cells in the bulge which act as repertoire of stem cells, are poorly characterized. In most cases, bulges are defined only by keratinocytes, which do not consider other cell populations in the region. This might explain that while bulge is important in hair regeneration (Miura et al., 2009), CD200^+^ population of bulge cells alone did not successfully induce hair follicle regeneration. Therefore, it might be essential to step back to the origin and consider the importance of CD200 in hair follicle regeneration, as it lacks *in vivo* evidence of the relationship between CD200 and hair follicle regeneration.

In this study, we attempted to determine whether CD200 is an important marker that identifies the hair regenerating portion of the hair follicle bulge cells. Both CD200^+^ and CD200^-^ bulge populations were collected and analyzed for their transcriptomic profiles and hair regenerative properties.

## 2 Methods

### 2.1 Ethical approval

For human samples, this study used leftover samples after surgical procedures. This study was approved by the Ethics Committee of Huashan Hospital of Fudan University (KY 2020-793) and Yokohama National University (Hitoi-2022-22). The patient provided informed consent to collect and use hair follicles damaged during transplantation. All animal experiments were approved by the Institutional Animal Care and Use Committee of Yokohama National University and conducted by following the ethical guidelines for medical and health research from the Ministry of Education, Culture, Sports, Science and Technology and the Ministry of Health, Labour and Welfare, Japan.

### 2.2 Immunofluorescence staining

Human scalp hair follicle were obtained from occipital region of patients with androgenic alopecia. Human hair follicle was fixed with 4% paraformaldehyde at room temperature overnight, followed by three washes with phosphate-buffered saline (PBS). The tissue was then sequentially placed in 15% and 30% sucrose solutions (Amresco, USA) for 1 day each, after which it was embedded in optimal cutting temperature compound (Leica, UK) and frozen for sectioning. Sections were cut at a thickness of 10 μm. Then, the cryosections were permeabilized and blocked at room temperature for 1 h with a solution containing 5% donkey serum and 0.1% Triton X-100 before incubation with primary antibodies against bulge cell markers, including mouse monoclonal antibody against human KRT15 (1 μg mL^-1^, Abcam, Cambridge, UK) and allophycocyanin(APC)-conjugated mouse monoclonal antibody against human CD200 (0.5 μg mL^-1^, eBioscience, San Diego, CA, USA) at 4 °C overnight. For samples incubated with KRT15 antibody, the sections were incubated with the corresponding secondary antibody at room temperature for 2 h, followed by incubation with 4′,6-diamidino-2-phenylindole (DAPI) (1 μg mL^-1^, Invitrogen, USA) for 10 min. For samples incubated with CD200 antibody, the sections were directly incubated with DAPI for 10 min. Images were acquired using a fluorescence microscope (Olympus, Tokyo, Japan).

### 2.3 Preparation of cells

The tissue at the bulge region of the hair follicle was isolated under a stereomicroscope (Nikon, Tokyo, Japan). The isolated bulge tissue was enzymatically dissociated into single-cell suspensions using a combination of dispase II (Gibco, Japan) and collagenase (Gibco, Japan). Cells were filtered through a 40 μm cell strainer (BD Falcon, New York, USA) to remove debris and the number of cells was counted for subsequent applications. For *in vitro* culture experiments, freshly isolated bulge cells were cultured under two different conditions, bulge cell medium 1 (BM1, ReMed Biotechnology, Shanghai, China) and bulge cell medium 2 (BM2, ReMed Biotechnology, Shanghai, China). Seeding the cells at a density of 2 × 10^4^ cells cm^-2^, cells were maintained at 37 °C in a humidified incubator with 5% CO_2_, and medium was refreshed every 2–3 days. When the cells reached 80%–90% confluence after 5–7 days of culture, they were harvested by treatment with TrypLE (Gibco, Denmark) for flow cytometry analysis, gene expression analysis, and *in vivo* transplantation experiments.

### 2.4 Fluorescence-activated cell sorting (FACS) and flow cytometry

The freshly isolated single-cell suspension of hair follicle bulge tissue was incubated with allophycocyanin (APC)-conjugated mouse monoclonal antibody against human CD200 (0.5 μg mL^-1^) at 4 °C for 30 min. The cells were then collected by centrifugation, rinsed with Dulbecco-modified phosphate-buffered saline without calcium and magnesium (D-PBS(−)), and suspended in D-PBS (−). Then, the cells were analyzed and sorted using a fluorescence-activated cell sorter (MA900, Sony, Tokyo, Japan). Both CD200^+^ and CD200^-^ cell populations were collected for subsequent analysis. The cells cultured under the two culture conditions, BM1 and BM2, were digested, rinsed and incubated with allophycocyanin (APC)-conjugated mouse monoclonal antibody against human CD200 (0.5 μg mL^-1^) and fluorescein isothiocyanate (FITC)-conjugated rat monoclonal antibody against integrin alpha 6 (ITGA6, 0.5 μg mL^-1^, eBioscience) at 4 °C for 30 min. After two washes with flow cytometry staining buffer (eBioscience), the cells were analyzed using a flow cytometer (CytoFLEX, A00-1-1102, Beckman Coulter, Brea, USA). The data were analyzed using CytExpert software (Beckman Coulter, Inc, 2.4.0.28).

### 2.5 Animal experiments

Embryonic dermal cells were isolated from C57BL/6J pregnant mice purchased from CLEA (Tokyo, Japan). The method was the same as that previously described (Yan et al., 2022). Then, 5 × 10^5^ sorted or cultured human hair follicle bulge cells were mixed with 5 × 10^5^ mouse embryonic dermal cells, resuspended in 50 µL of Dulbecco’s Modified Eagle Medium (DMEM, Gibco, China) containing 10% fetal bovine serum (Sigma-Aldrich, St. Louis, MO, USA), and injected into the dorsal skin of nude mice (5-week-old ICR nude mice, Charles River, Kanagawa, Japan) using a 1 mL syringe to perform the patch assay. Transplantation was conducted under anaesthesia with 2% isoflurane at 0.5 L min^-1^. The mice were euthanized by cervical dislocation 3 weeks after transplantation. Skin samples were collected, and images were acquired using a stereomicroscope (Olympus, Tokyo, Japan). The tissues were enzymatically digested with collagenase solution, and the number of hairs was manually counted under a stereomicroscope.

### 2.6 Gene expression analysis

Total RNA was extracted from freshly isolated or cultured cells using a commercial RNA isolation kit (Qiagen, Hilden, Germany), and cDNA was synthesized via reverse transcription using the ReverTra Ace™ RT-qPCR kit (Toyobo, Osaka, Japan) according to the manufacturer’s instructions.

Quantitative polymerase chain reaction was performed using SYBR Premix Ex Taq II (Takara Bio, Shiga, Japan) on the StepOne Plus RT-PCR system (Applied Biosystems 7500 Real-Time PCR System, Thermo Fisher Scientific, Waltham, MA, USA). The primers listed in Table 1 were used to assess the expression levels of the following genes: CD200, KRT15, KRT19, LGR5, LIM homeobox 2 (LHX2), and SOX9. Relative gene expression of all genes was measured relative to expression of glyceraldehyde 3-phosphate dehydrogenase (GAPDH) and analyzed using the ΔC_T_ method.

**TABLE 1:** Sequence of primers used for polymerase chain reaction.

Genes		Sequence of primers (5´→3′)
GAPDH	Forward	GGA​GCG​AGA​TCC​CTC​CAA​AAT
	Reverse	GGC​TGT​TGT​CAT​ACT​TCT​CAT​GG
CD200	Forward	TGA​CTC​TGT​CTC​ACC​CAA​ATG
	Reverse	GCT​TAG​CAA​TAG​CGG​AAC​TG
K15	Forward	GAC​GGA​GAT​CAC​AGA​CCT​GAG
	Reverse	CTC​CAG​CCG​TGT​CTT​TAT​GTC
SOX9	Forward	AGC​GAA​CGC​ACA​TCA​AGA​C
	Reverse	CTG​TAG​GCG​ATC​TGT​TGG​GG
LHX2	Forward	ATG​CTG​TTC​CAC​AGT​CTG​TCG
	Reverse	GCA​TGG​TCG​TCT​CGG​TGT​C
K19	Forward	AAC​GGC​GAG​CTA​GAG​GTG​A
	Reverse	GGA​TGG​TCG​TGT​AGT​AGT​GGC
LGR5	Forward	CAC​CTC​CTA​CCT​AGA​CCT​CAG​T
	Reverse	CGC​AAG​ACG​TAA​CTC​CTC​CAG

### 2.7 Transcriptomic analysis by bulk RNA-sequencing

To conduct bulk RNA sequencing, total RNA was extracted with TRIzol reagent (Invitrogen, CA, USA) according to the manufacturer’s protocol. RNA integrity was assessed using the Agilent 2100 Bioanalyzer (Agilent Technologies, Santa Clara, CA, USA). Then the libraries were constructed using VAHTS Universal V10 RNA-seq Library Prep Kit (Premixed Version) according to the manufacturer’s instructions. The libraries were sequenced on an DNBSEQ-T7 platform and 150 bp paired-end reads were generated. Raw reads of fastq format were firstly processed using fastp and the low-quality reads were removed to obtain the clean reads. Then, the data was normalised for further analysis.

### 2.8 Single-cell RNA-sequencing analysis

Hair follicle bulges were digested into a single-cell suspension. An scRNA-seq library was prepared using the DNBelab C Series Single-Cell Library Prep set. Single-cell suspensions were converted to barcoded scRNA-seq libraries through droplet encapsulation and emulsion breakage. After droplet breakage, the beads underwent chemical conversion via the on-bead IAA reaction. The beads were then washed once with 1× SSC, followed by reverse transcription, enzyme digestion, second-strand synthesis, PCR amplification of the cDNA library, and indexed sequencing libraries according to the manufacturer’s protocol. The final libraries were sequenced using an MGI DNBSEQ-T7 sequencer. DNBC4tools was used for data preprocessing, and subsequent analysis was conducted using Seurat v5. The data have been deposited at China National Center for Bioinformation BioProject repository (accession number PRJCA060736). 

### 2.9 Statistical analysis

All quantitative data are presented as mean ± standard deviation unless otherwise mentioned. Comparisons were analyzed using one-way analysis of variance (ANOVA) followed by Tukey’s honestly significant differential tests. Statistical significance was set at *p* < 0.05.

## 3 Results

### 3.1 Identification of hair follicle bulge from human hair follicle

**FIGURE 1:**
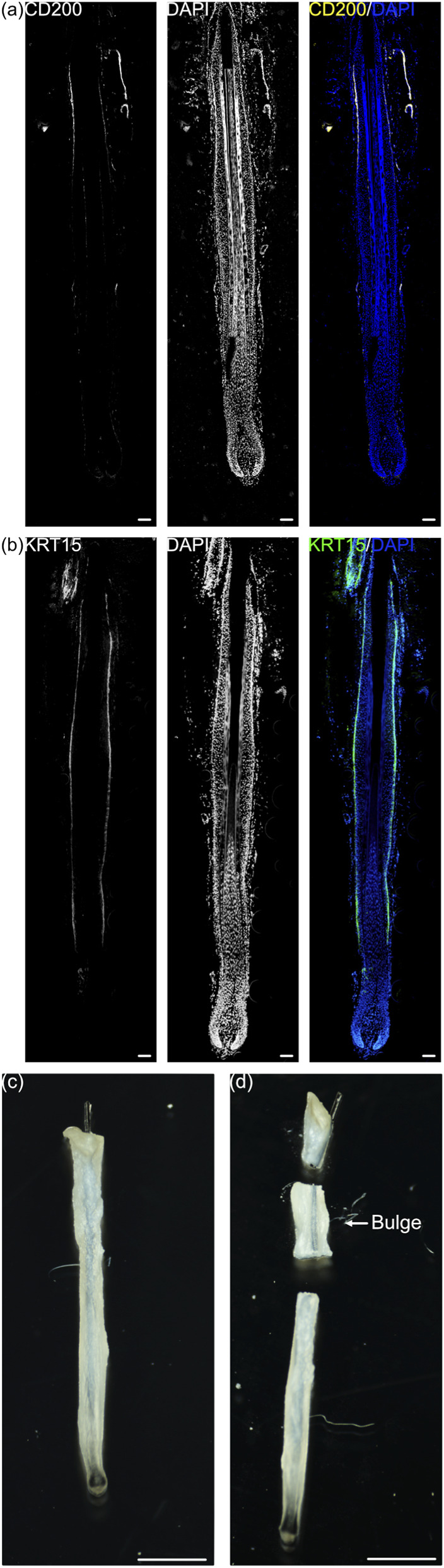
Identification of hair follicle bulge. Immunofluorescence staining of longitudinal section of anagen hair follicle for **(a)** CD200 and **(b)** KRT15. The scale bars are 100 μm. A CD200^+^ KRT15^+^ portion of the hair follicle was identified by the distance from the interfollicular epidermis. Then, from **(c)** intact hair follicle, **(d)** the portion was dissected and isolated. The scale bars are 1 mm.

To accurately identify the location of the human hair follicle bulge, we examined the expression of CD200 and KRT15, which are both reported markers for hair follicle bulge in anagen hair follicles (Inoue et al., 2009). Immunofluorescence staining of longitudinal sections of anagen hair follicles revealed a distinct portion of the hair follicle where both CD200 and KRT15 were expressed. Compared to CD200 (Figure 1a), KRT15 is widely distributed across the hair follicle, extending from the hair follicle bulge to the hair follicle bulb (Figure 1b). In this study, the region where CD200^+^ cells were localized was defined as the hair follicle bulge. The distance from the interfollicular epidermis to the top of the CD200^+^ region and the length of the CD200^+^ region were measured. These parameters were used as a reference to isolate the hair follicle bulge for subsequent studies (Figures 1c,d).

### 3.2 Single-cell RNA-sequencing revealed variation of cells in hair follicle bulge

As discussed in a previous study, the hair follicle bulge is defined by its structure (Ohyama, 2007), but not by a specific type of cells. Therefore, it is possible that a variety of cells expressing KRT15 and KRT14 are present in the hair follicle bulge. To identify the population of cells in the hair follicle bulge, the bulge, as defined in Figure 1d, was isolated and digested into a single-cell suspension. The cell suspension was subjected to single-cell RNA sequencing.

**FIGURE 2:**
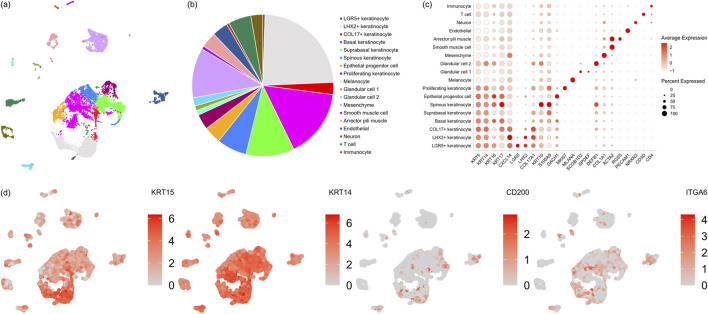
Single cell transcriptomic analysis of hair follicle bulge cells. **(a)** UMAP plot of hair follicle bulge cells. **(b)** Proportion of each population of cells in hair follicle bulge. **(c)** Dot plot representation of some known marker genes of cells in hair follicle bulge. **(d)** Expression of known marker genes in each cluster of cells.

Eighteen clusters were identified from the UMAP plot obtained (Figure 2a). Among these clusters, eight clusters were hair follicle keratinocytes, which comprised 68.13% of the cells (Figure 2b). Other populations included mesenchyme (11.41%), T cells (5.18%), arrector pili muscle (3.55%), endothelial cells (3.61%), glandular cells (2.95%), immunocytes (2.72%), melanocytes (1.13%), smooth muscle cells (0.72%) and neurons (0.61%). Each cell population was defined by its known marker genes (Figure 2c), and cells from all clusters expressed KRT14 and KRT15. CD200 and ITGA6 were expressed in only a portion of the cells (Figure 2d).

### 3.3 Hair regenerative capability of CD200^+^ and CD200^-^ subpopulations in hair follicle bulge

**FIGURE 3:**
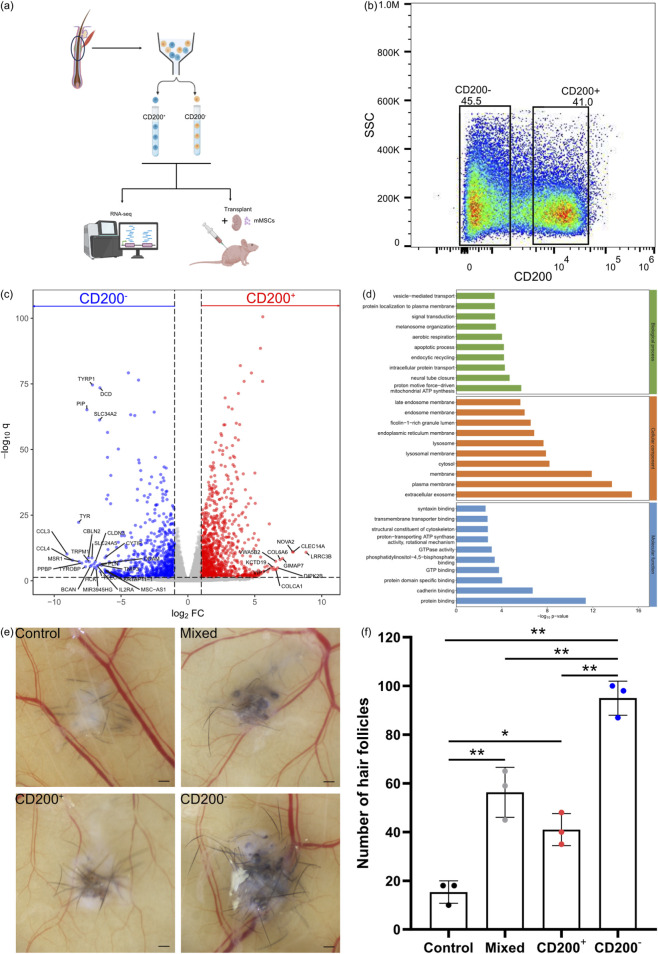
Hair regenerative capability of CD200^+^ and CD200^-^ hair follicle bulge cell. **(a)** Schematic diagram of the experimental procedure. **(b)** Fluorescence-activated cell sorting of CD200^+^ and CD200^-^ hair follicle bulge cells from the bulge region of fresh hair follicles. **(c)** Differentially expressed genes from RNA-seq of sorted CD200^+^ and CD200^-^ hair follicle bulge cells. **(d)** Gene ontology analysis of CD200^+^ and CD200^-^ hair follicle bulge cells. **(e)** Representative photographs of hair follicle generated from CD200^+^ and CD200^-^ hair follicle bulge cells after patch assay. The scale bars are 500 μm. **(f)** Number of hair follicles generated from CD200^+^ and CD200^-^ hair follicle bulge cells after patch assay. Each plot represents three biological replicates. Mean ± standard deviation. Statistical significance was analyzed by one-way ANOVA followed by Tukey’s HSD *post hoc* test. ∗*p* < 0.05, ∗∗*p* < 0.01.

CD200 is believed to be a crucial marker of hair follicle bulge cells. However, the importance of CD200 in hair follicle induction remains unknown. To validate the importance of CD200 in hair follicle bulge cell, we sorted hair follicle bulge cells into CD200^+^ and CD200^-^ fractions (Figures 3a,b), and the cells were analyzed using whole transcriptomic analysis. The CD200^-^ fraction was enriched with genes related to melanin biosynthesis and immunomodulation, whereas the CD200^+^ fraction was enriched with genes related to gene regulation (Figure 3c). Gene ontology analysis suggested that CD200^-^ fractions were enriched with genes related to signal transduction, particularly vesicle-mediated transport, protein localization, signal transduction, and intracellular protein transport, suggesting that the cells were engaged in active signalling (Figure 3d).

Patch assays were conducted to investigate the hair inductive capability of the CD200^+^ and CD200^-^ fractions of hair follicle bulge cells. As shown in Figure 3e, the hair follicles induced by the CD200^-^ fraction were thicker than those induced by the CD200^+^ fraction, and the number of hair follicles was higher (Figure 3f).

### 3.4 Single-cell transcriptomic analysis revealed the proportion of cells in CD200^-^ hair follicle bulge cells

**FIGURE 4:**
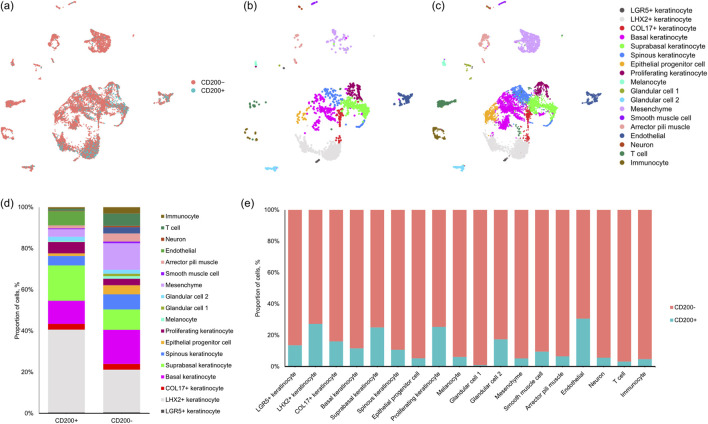
Composition of bulge cells defined by CD200 expression. **(a)** UMAP plot of the data obtained from single cell transcriptomic analysis and grouped by CD200 expression. UMAP plot of **(b)** CD200^+^ and **(c)** CD200^-^ hair follicle bulge cells. **(d)** Proportion of hair follicle bulge cells in each subpopulation. **(e)** Fraction of each cluster in each subpopulation of hair follicle bulge cells.

To investigate the composition of cells in each fraction of hair follicle bulge cells, CD200^+^ (where CD200 expression >0) and CD200^-^ cells (where CD200 expression = 0) in hair follicle bulge were subclustered from data obtained from scRNA-seq (Figures 4a–c). The results suggested that a higher number of cells were identified in the CD200^-^ subpopulation. Among the 11,712 sequenced cells, 9,826 cells were clustered in the CD200^-^ subpopulation, while only 1,886 cells were clustered in the CD200^+^ cells. Comparing the ratio of cells, the ratio of glandular cell 1 was the highest in CD200^-^ cells against CD200^+^ cells, which was 119-fold increased. The rank is followed by T cells and immunocytes, which would reconstitute the niche for the growth of hair follicles, mesenchyme, and epithelial progenitor cells, which are important components of hair follicles. Neurons and arrector pili muscles were also enriched in the CD200^-^ fraction, which provides factors that stimulate hair follicle regeneration. In contrast, the CD200^+^ subpopulation is enriched with a higher fraction of quiescent hair follicle bulge cells, which express LHX2 (Figures 4d,e).

### 3.5 Hair regenerative capability of cultured CD200^+^ and CD200^-^ hair follicle bulge cells

**FIGURE 5:**
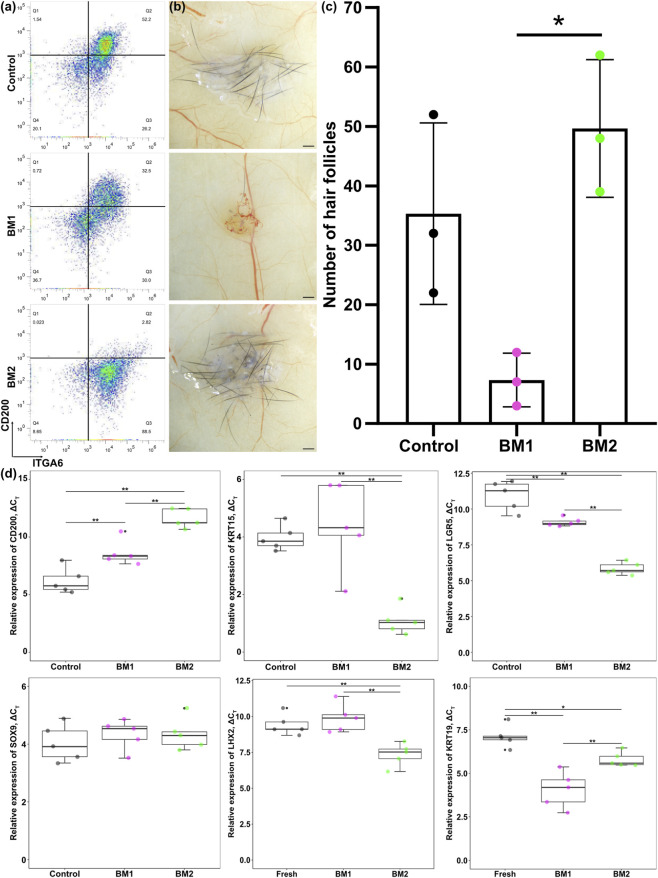
Hair regenerative ability of cultured CD200^+^ and CD200^-^ fractions of human hair follicle bulge cells. **(a)** Flow cytometric analysis of hair follicle bulge cells cultured in BM1 and BM2. **(b)** Representative photographs of hair follicles generated from cultured CD200^+^ and CD200^-^ hair follicle bulge cells. **(c)** Number of hair follicles generated from cultured CD200^+^ and CD200^-^ hair follicle bulge cells after patch assay. Each plot represents three biological replicates. **(d)** Relative expression of CD200, KRT15, LGR5, SOX9, LHX2, and KRT19 between cultured CD200^+^ and CD200^-^ hair follicle bulge cells. ∗*p* < 0.05, ∗∗*p* < 0.01.

To investigate whether cultured bulge cells exhibit a similar phenomenon to non-cultured hair follicle bulge cells, we cultured bulge cells using two distinct hair follicle epithelial cell culture systems: BM1 and BM2. The two culture systems distinctly regulate the expression of CD200 in cultured hair follicle bulge cells. Flow cytometric analysis suggested that all groups were ITGA6^+^ cells, which is also a marker for hair follicle bulge cells. In contrast, only the control and cells cultured with BM1 were CD200^+^ (Figure 5a). Similar to the results obtained with non-cultured hair follicle bulge cells, cells cultured with BM2 (CD200^-^) exhibited higher hair follicle regenerative capability (Figures 5b,c). Further analysis using reverse transcriptase-quantitative polymerase chain reaction suggested that cells cultured with BM2 expressed higher mRNA levels of KRT15, LGR5 and LHX2 than those cultured with BM1 (Figure 5d).

**FIGURE 6:**
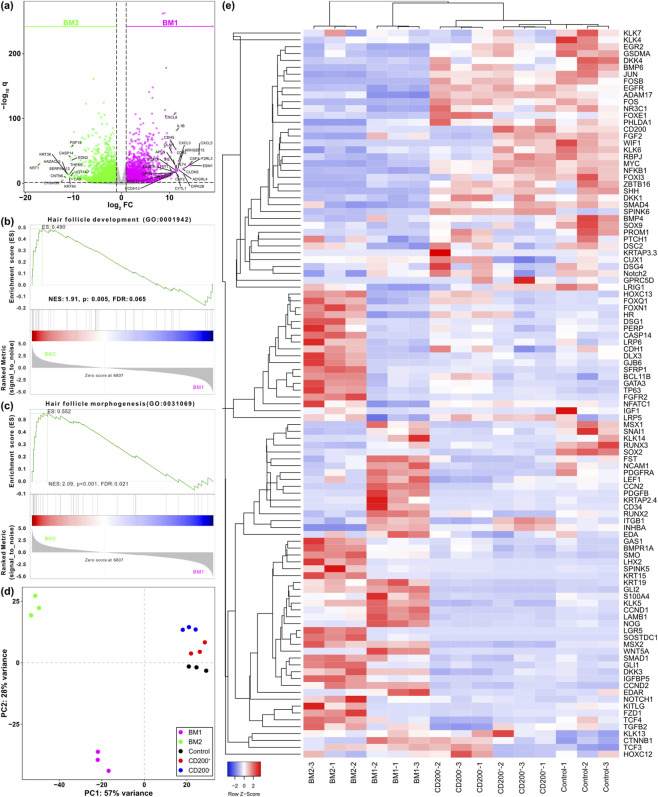
Transcriptomic analysis of non-cultured and cultured human hair follicle bulge cells. **(a)** Differentially expressed genes from RNA-seq of cultured CD200^+^ and CD200^-^ hair follicle bulge cells. **(b,c)** Gene set enrichment analysis of cultured CD200^+^ and CD200^-^ hair follicle bulge cells. **(d)** Principal component analysis of non-cultured and cultured human hair follicle bulge cells. **(e)** Heatmap plotting expression of genes in hair follicle development pathway between non-cultured and cultured bulge cells.

To obtain deeper insights into the properties of cells cultured with BM1 and BM2, the transcriptomic profiles of these cells were evaluated using RNA sequencing. Comparing cells cultured with BM1 against cells cultured with BM2, we observed higher expression of genes related to hair follicle regeneration, such as endothelin 2, fibroblast growth factor, and various types of cytokeratin in cells cultured with BM2, whereas genes related to inflammatory responses were enriched in cells cultured with BM1 (Figure 6a). Gene set enrichment analysis of the data obtained from cells cultured with BM1 and BM2 suggested that genes related to hair follicle development and hair follicle morphogenesis were enriched in cells cultured with BM2 (Figures 6b,c), further suggesting that the CD200^+^ fraction might have lower hair regenerative capability.

Dimensional reduction by principal component analysis showed distinct clusters of both BM1 and BM2 compared to non-cultured cells (Figure 6d), and these cells exhibit distinct transcriptional profiles in the hair follicle development pathway (Figure 6e). Cells cultured with BM2 not only exhibited higher expression levels of genes related to hair follicle stem cells, such as LGR5 and KRT15, but also various genes related to the development of hair follicles compared to other groups.

## 4 Discussion

The hair follicle bulge is defined by its structure. This structure is permanent regardless of the stages of the hair cycle and houses various types of stem cells, making it the repertoire of stem cells in human skin. The stem cells contained in the hair follicle bulge vary, and the expression of CD200 is often used as a key marker for defining hair follicle bulge cells. However, the direct contribution of CD200 expression to the hair follicle regenerative capacity has not been systematically investigated.

Through immunofluorescence staining, we identified the location of the hair follicle bulge relative to interfollicular epidermis (Figures 1a,b). This portion of the hair follicle was extracted (Figure 1d), and the cells were analyzed. In contrast to the common annotation that regards bulges as keratinocytes, 18 clusters of cells were identified, among which 8 clusters were hair follicle keratinocytes (Figure 2b). Cells from all clusters expressed KRT14 and KRT15 (Figure 2d), which may have resulted in annotation of these cells as hair follicle keratinocytes in previous studies. These cells were virtually separated into CD200^+^ fraction and CD200^-^ fraction based on their CD200 expression. Based on the distribution of CD200 expression (Supplementary Figure S1), the peaks were located at CD200 expression = 0 and CD200 expression = 1. A distinct gap existed in the distribution. Therefore, it is appropriate to divide the cell population into CD200^+^ and CD200^-^ population based on CD200 expression = 0 and CD200 expression >0. Between the 2 fractions, CD200^-^ fraction was enriched with a variety of cells, including glandular cells, mesenchymal cells, and immunocytes. The markers from part of these cells were captured by bulk RNA-seq and enriched in top 20 genes in CD200^-^ fraction (Table 2). These cells produce extracellular matrices and factors, such as laminin, collagen, desmosomes, epidermal growth factor, CD39, activin, non-canonical Wnt, and neuregulin, which may contribute to hair follicle regeneration. The results obtained from the patch assay further confirmed that the CD200^-^ fraction exhibited higher hair regenerative capability than the CD200^+^ fraction (Figure 3e), suggesting that CD200 may not be necessary for hair follicle regeneration. This phenomenon was further validated using cultured hair follicle bulge cells (Figure 5c).

**TABLE 2:** Top 20 regulated genes in CD200^+^ and CD200^-^ fraction of hair follicle bulge which define their identity.

CD200^-^ fraction	CD200^+^ fraction
CCL3	LRRC3B
CCL4	CLEC14A
TYR	NOVA2
MSR1	GIMAP7
PPBP	COL6A6
TYROBP	DIPK2B
LOC105375436	COLCA1
PIP	VWA5B2
CBLN2	LOC105374309
TRPM1	KCTD19
BCAN	LOC101928636
LOC105373116	VIP
SLC24A5	NKX1-1
TYRP1	LOC107985238
LINC00518	DELEC1
HCK	HOXD9
CLDN3	USHBP1
MIR3945HG	C12orf50
IL2RA	LOC105373545
PLN	LOC101928373

As suggested by previous study, CD200 might function as a marker of immune privilege, suppressing immune activation via CD200-CD200 receptor interaction (Rosenblum et al., 2006). In our study, we have also identified the immune privilege of CD200^+^ fraction based on data obtained from scRNA-seq (Supplementary Figure S2a). This result validates the observation in previous study. On the other hand, while CD200^-^ fraction exhibit higher hair follicle regenerative capability, it has higher quiescence score compared to CD200^+^ fraction (Supplementary Figure S2b) and the cell cycle scoring was similar for both fractions, suggesting that the hair regenerative capability might be irrelevant of quiescence state or cell cycle.

Comparing the data obtained from RNA-seq for non-cultured and cultured CD200^-^ fractions showed that the correlation score was between 0.55–0.60 between the 2 conditions (Supplementary Figure S3), suggesting moderate similarity between both groups. Interestingly, both non-cultured and cultured CD200^-^ fractions exhibited higher expression of genes in the forkhead box gene family. These genes regulate various processes, including stem cell proliferation, differentiation, and hair follicle morphogenesis (Brancaccio et al., 2004; Cai et al., 2009; Zhao et al., 2015). Higher expression of these genes in CD200^-^ fractions suggests active regulation of hair follicle regeneration in CD200^-^ fractions of hair follicle bulge cells.

In our *in vitro* expansion experiments, two cultured systems that could modulate CD200 expression differently were used. Notably, culture conditions that maintained high CD200 expression did not support superior hair regeneration *in vivo*. In contrast, culture systems associated with reduced CD200 expression selectively enriched epithelial morphogenesis and differentiation-related genes, resulting in enhanced regenerative outcomes in the cells. These findings suggest that *in vitro* culture reshapes the state of bulge cells and selectively amplifies specific functional cell populations. Therefore, decreased CD200 expression under certain culture conditions may be correlated with a transition toward a more regenerative epithelial state rather than representing a loss of bulge cell identity.

Flow cytometric analysis was performed to rule out the effect of contamination by basal keratinocytes from mouse skin when isolating embryonic dermal cells. The fraction of cells expressing KRT14, which is a key marker of basal keratinocytes was almost equal to the readout obtained from the isotype control (Supplementary Figure S4), suggesting that contamination was negligible. Therefore, the hair follicles generated in our patch assay might not be induced by mouse epidermal cells.

In this study, we found that CD200 may not be necessary for hair follicle regeneration within the hair follicle bulge region. Other functional cell populations exist beyond those traditionally defined by CD200 expression. Our results suggest that hair follicle regenerative capacity may rely on multiple cell types and the niche they establish, rather than being attributed to a single subpopulation defined by a specific surface marker.

This study provided useful insight to the development of cell-based therapy for hair follicle regeneration. CD200^-^ population in bulge, but not CD200^+^ population exhibit enhanced hair follicle regenerative capacity, which could potentially provide hints to the future clinical study of hair follicle regeneration. In addition, successful expansion of hair follicle bulge in BM2 culture system while maintaining its capability in hair follicle regeneration demonstrating its potential in in vitro expansion of hair follicle derived cells for hair follicle regeneration. Combining the cells cultured with BM2 with other cells which contribute to hair follicle regeneration, e.g., active hair follicle dermal papilla cells might provide the possibility for clinical hair follicle regeneration.

Nevertheless, this study has some limitations. It remains unclear which population of cells in the CD200^-^ fraction or their combination is the functioning population related to hair follicle regeneration. A detailed study is being conducted to identify the populations related to hair follicle regeneration, and the relationship between the CD200^+^ and CD200^-^ fractions is being investigated.

## Data Availability

All data needed to evaluate the conclusions are present in this article. Other materials are available from corresponding author upon reasonable request.
