# Supplementary material for: Regenerative potential of CD200- subpopulations of hair follicle bulge
Source: Front Cell Dev Biol. 2026 Apr 23;14:1808471. doi: 10.3389/fcell.2026.1808471 (PMC13149264; doi:10.3389/fcell.2026.1808471)
Supplement: Supplementary file 1 [file DataSheet1.pdf]

Supplementary Material

Regenerative Potential of CD200^{-} Subpopulations of Hair Follicle Bulge

Ting Sun[1,2], Sayuri Hamano[1], Jing Chen[2], Lichen Ling[2], Jinran Lin[3], Lei Yan[1,4], Tatsuto Kageyama[1,4], Fuyue Wu[2], Zheng Lin Tan[2*], Wenyu Wu[3*], Junji Fukuda[1,4*]

[1]Faculty of Engineering, Yokohama National University, 79-5 Tokiwadai, Hodogaya-ku, Yokohama, Kanagawa 240-8501, Japan

[2]ReMed Regenerative Medicine Clinical Application Institute, Shanghai, People's Republic of China

[3]Department of Dermatology, Huashan Hospital of Fudan University, Shanghai, People's Republic of China

[4]Kanagawa Institute of Industrial Science and Technology, 3-25-22 Tonomachi, Kawasaki, Kanagawa 210-0821, Japan.

*** Correspondence:**
Junji Fukuda (Academic)
fukuda@ynu.ac.jp

Wenyu Wu (Medical)
wuwenyu@huashan.org.cn

Zheng Lin Tan (Technical)
zhenglin.tan@remed-bio.com

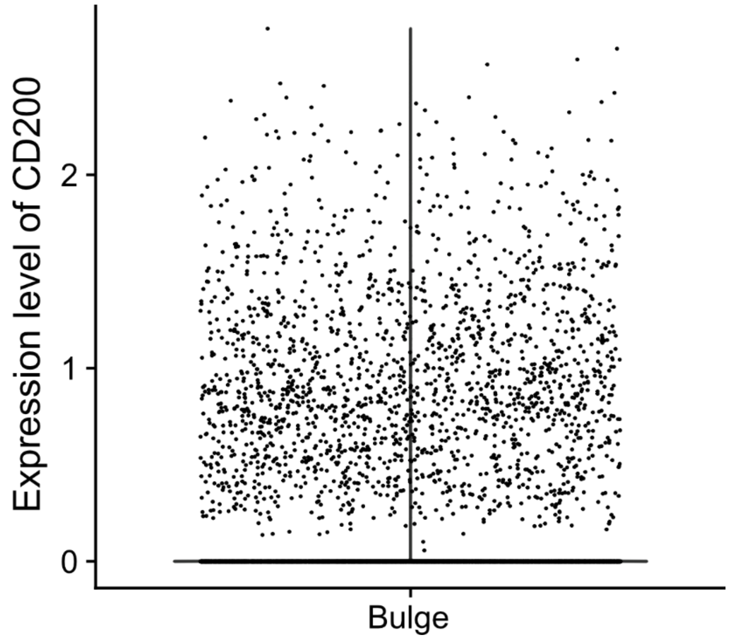

Figure S1 Expression level of CD200 of all cells analyzed by scRNA-seq.

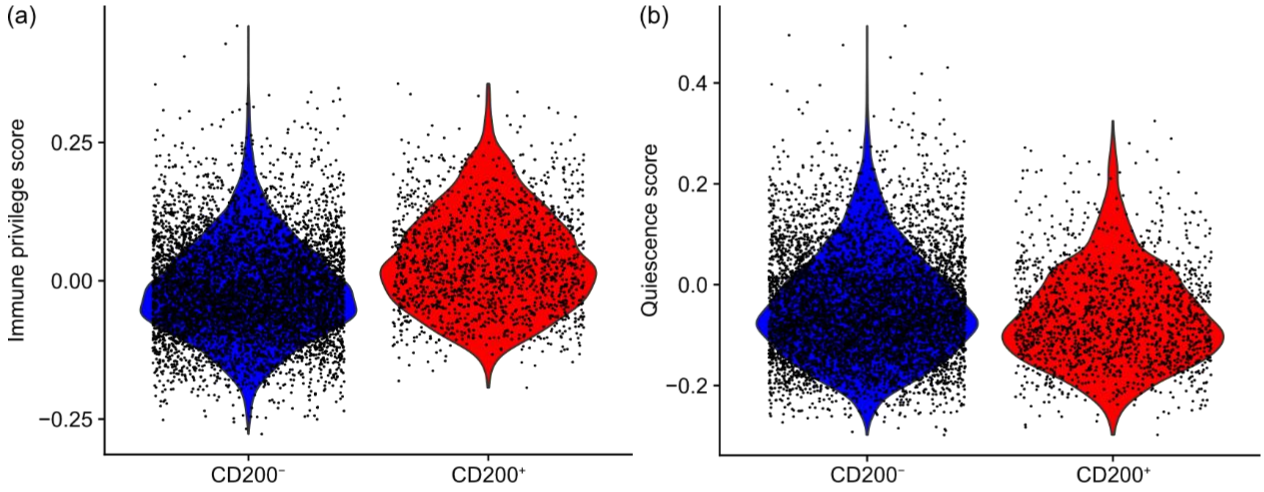

Figure S2 Analysis of CD200$^+$ fraction and CD200$^-$ fraction of data obtained from scRNA-seq based on scoring method. (a) Immune privilege score and (b) stem cell quiescence score of CD200$^+$ and CD200$^-$ fraction.

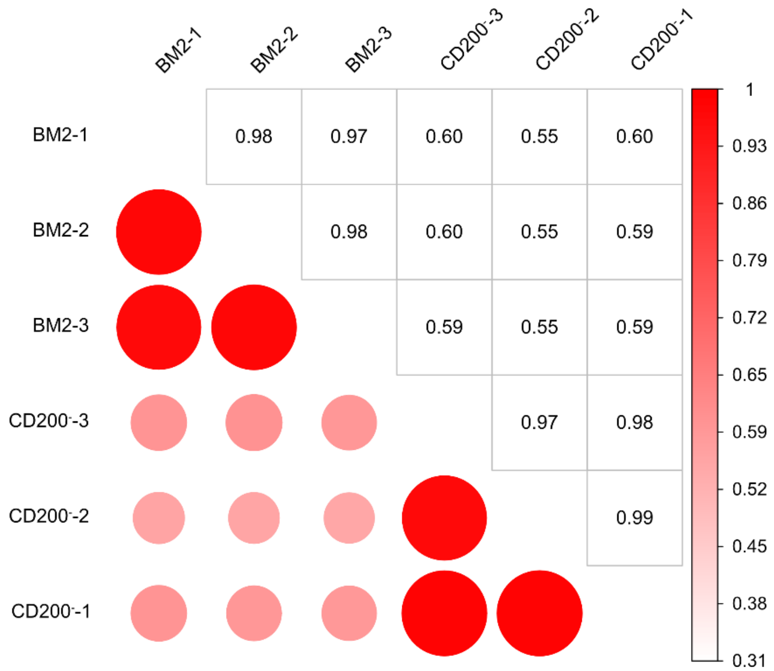

Figure S3 Correlation matrix of the data obtained from RNA-seq for cells cultured with BM2 and CD200⁻ fraction isolated from bulge.

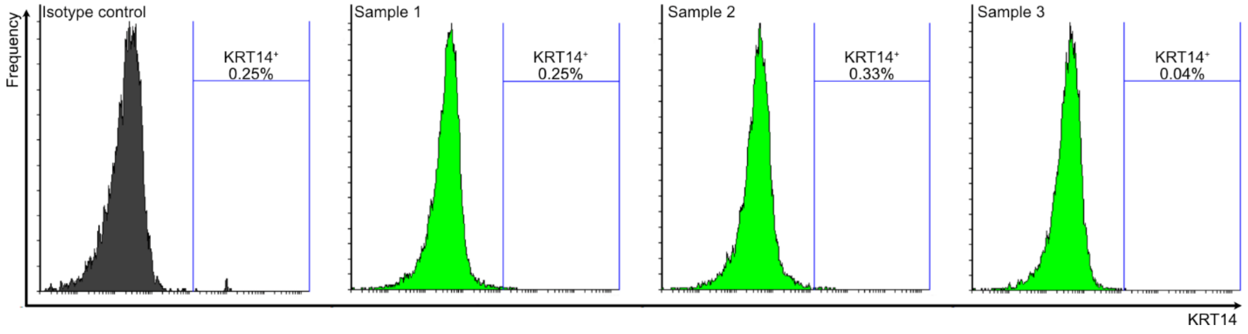

Figure S4 Histogram of flow cytometric analysis of the expression of KRT14 in mouse embryonic dermal cells. The number in each subplot indicates percentage of cells in the gated region.
